# The immunoproteasome and viral infection: a complex regulator of inflammation

**DOI:** 10.3389/fmicb.2015.00021

**Published:** 2015-01-29

**Authors:** Mary K. McCarthy, Jason B. Weinberg

**Affiliations:** ^1^Department of Microbiology and Immunology, University of MichiganAnn Arbor, MI, USA; ^2^Department of Pediatrics and Communicable Diseases, University of MichiganAnn Arbor, MI, USA

**Keywords:** proteasome, immunoproteasome, viral pathogenesis, CD8, T cell, NF-κB

## Abstract

During viral infection, proper regulation of immune responses is necessary to ensure successful viral clearance with minimal host tissue damage. Proteasomes play a crucial role in the generation of antigenic peptides for presentation on MHC class I molecules, and thus activation of CD8 T cells, as well as activation of the NF-κB pathway. A specialized type of proteasome called the immunoproteasome is constitutively expressed in hematopoietic cells and induced in non-immune cells during viral infection by interferon signaling. The immunoproteasome regulates CD8 T cell responses to many viral epitopes during infection. Accumulating evidence suggests that the immunoproteasome may also contribute to regulation of proinflammatory cytokine production, activation of the NF-κB pathway, and management of oxidative stress. Many viruses have mechanisms of interfering with immunoproteasome function, including prevention of transcriptional upregulation of immunoproteasome components as well as direct interaction of viral proteins with immunoproteasome subunits. A better understanding of the role of the immunoproteasome in different cell types, tissues, and hosts has the potential to improve vaccine design and facilitate the development of effective treatment strategies for viral infections.

## INTRODUCTION

The host outcome of viral infection depends on the successful balance of immune responses that contribute to control of viral replication, but those responses may also mediate aspects of host tissue damage that accompany infection. Antibodies, cytokines, CD4 T cells, and CD8 T cells can all play important roles in controlling viral replication and viral clearance from the host. CD8 T cells kill infected target cells by two major pathways: perforin/granzyme-mediated pathways and Fas–Fas ligand (FasL)-mediated pathways (Russell and Ley, 2002; Hoves et al., 2010). CD8 T cells can also contribute to elimination of infected cells through production of antiviral cytokines, such as IFN-γ and TNF-α. A number of viruses are eliminated at least partially through CD8 T cell-dependent mechanisms in mouse models, including lymphocytic choriomeningitis virus (LCMV), influenza virus, the mouse-specific orthopoxvirus ectromelia virus, and murine gammaherpesvirus (MHV-68; Ehtisham et al., 1993; Kägi et al., 1994; Walsh et al., 1994; Müllbacher et al., 1999). However, CD8 T cells can also contribute to CNS, liver, and cardiac pathology during infection with LCMV and coxsackievirus B3 (Buchmeier et al., 1980; Henke et al., 1995; Lang et al., 2008).

**FIGURE 1:**
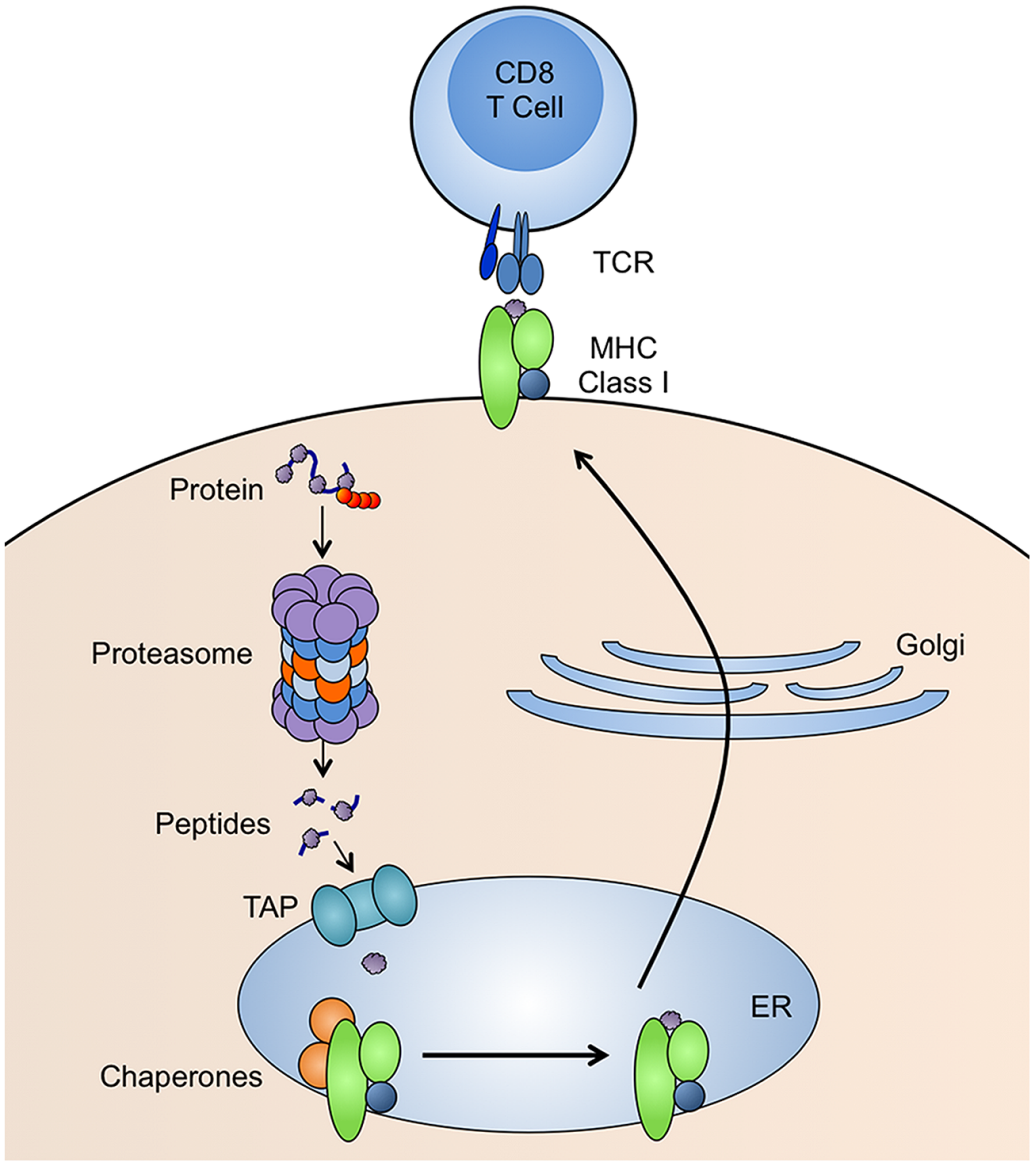
**MHC class I antigen presentation pathway.** Proteins with ubiquitin tags (red spheres) are degraded by proteasomes and the resulting peptides are transported into the endoplasmic reticulum (ER) by TAP. In the ER, the peptide is loaded onto MHC class I molecules by many molecular chaperones. The peptide-MHC class I complex is then transported to the cell surface for presentation to CD8 T cells.

CD8 T cells recognize peptides bound to MHC class I molecules. The generation of peptide-MHC class I complexes involves many steps (**Figure 1**). Peptides that bind tightly to MHC class I are 8–11 amino acids in length and have anchor residues, which are generally in the C-terminus, but can be present elsewhere in the peptide sequence (Falk et al., 1991). The vast majority of these peptides are generated by proteasomes (Rock et al., 1994), although extended versions of peptides produced by the proteasome can be trimmed by aminopeptidases in the cytosol (Stoltze et al., 2000) or endoplasmic reticulum (ER; Snyder et al., 1994; Craiu et al., 1997a). Peptides are transported from the cytoplasm to the ER by an ER-resident heterodimeric protein called transporter for antigen processing (TAP; Neefjes et al., 1993). TAP is a subunit of the MHC class-I-loading complex, a ~1 MDa complex within the ER, that clusters the molecules involved in MHC class I loading in order to increase the efficiency of the process. Once a peptide is successfully bound, the MHC class I molecule is released from the MHC class-I-loading complex and delivered to the cell surface for presentation to CD8 T cells.

Activation of CD8 T cells depends on the proteasome for generation of antigenic peptides presented on MHC class I molecules on the surface of either infected cells or antigen presenting cells (APCs). The immunoproteasome is a specialized type of proteasome with altered peptide cleavage properties that is constitutively expressed in hematopoietic cells and induced in non-immune cells under conditions of inflammation. Evidence suggests that the immunoproteasome may play an important role during viral infection through regulation of CD8 T cell responses and proinflammatory cytokine production, activation of the NF-κB pathway, and management of oxidative stress (Groettrup et al., 2009; Ebstein et al., 2012; Basler et al., 2013b; Warnatsch et al., 2013).

## STANDARD PROTEASOMES AND IMMUNOPROTEASOMES

**FIGURE 2:**
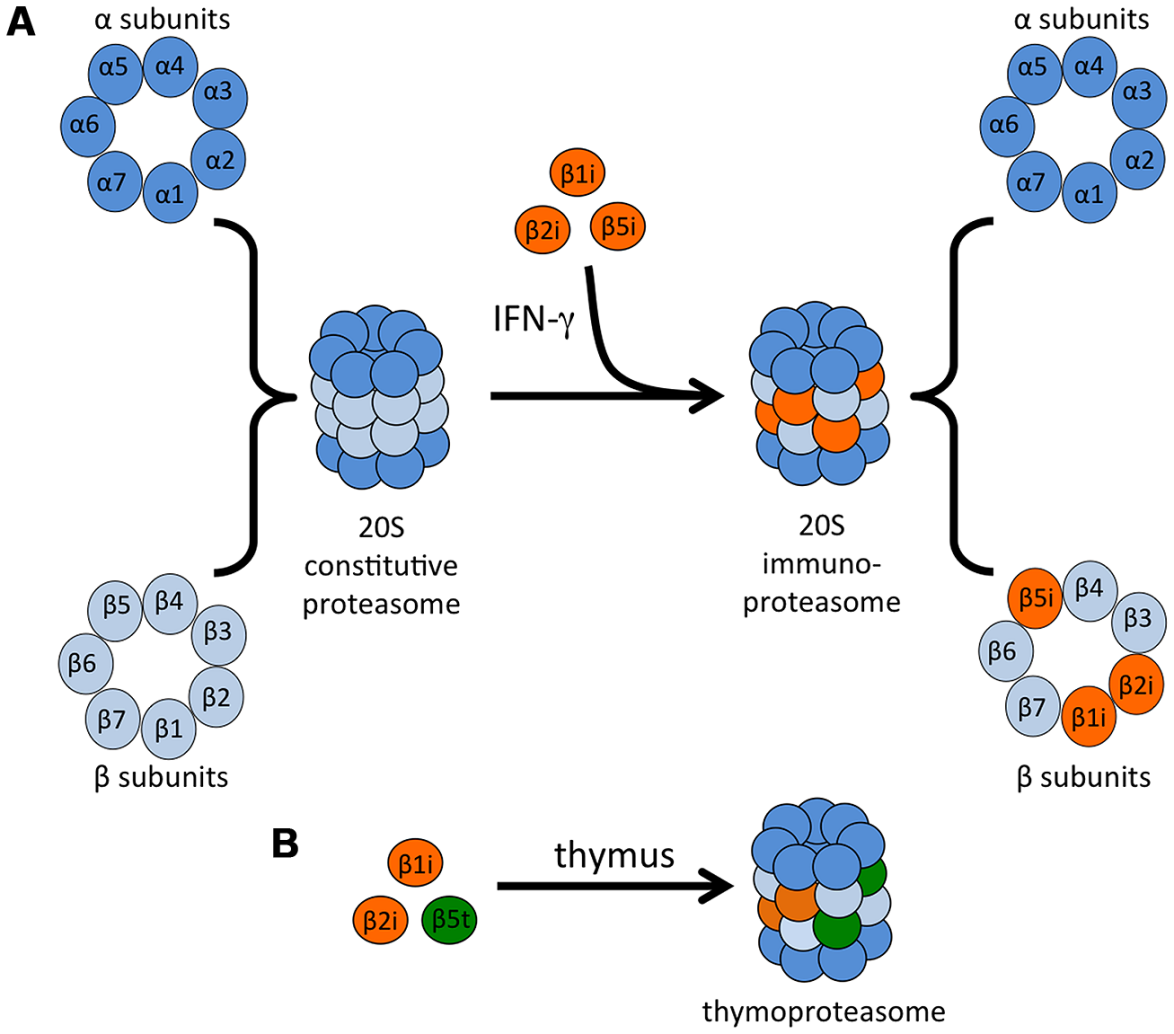
**Immunoproteasome formation and the thymoproteasome. (A)** The catalytic core of the 20S proteasome is comprised of two outer α rings and two inner β rings. IFN-γ exposure induces the synthesis of three β “immunosubunits,” which are incorporated into newly formed proteasomes in place of their constitutive counterparts to form the 20S immunoproteasome. **(B)** In the thymus, a specialized type of proteasome is expressed in cTECs. This proteasome contains the immunosubunits β1i and β2i as well as a cTEC-specific proteasome subunit β5t.

Proteasomes are large complexes responsible for the regulated degradation of almost all cellular proteins, and as such proteasome activity is required for cell viability (Rock et al., 1994; Tanaka, 1995). Proteasomes also play a primary role in the generation of antigenic peptides for presentation on MHC class I molecules, but not on MHC class II (Rock et al., 1994; Groettrup et al., 1996c; Craiu et al., 1997b). The 20S proteasome core is a barrel-shaped complex that is composed of four stacked heptameric rings: two outer alpha rings and two inner beta rings (**Figure 2A**; Groll et al., 1997; Unno et al., 2002). The proteasome may be associated with activator caps, discussed below. The catalytic activity is restricted to three of the beta subunits, β1 (also called Y in vertebrates), β2 (Z), and β5 (X), that account for the caspase-like, trypsin-like, and chymotrypsin-like activities of the proteasome, respectively (DeMartino and Slaughter, 1999). The active sites of each of these proteins face toward the lumen of the proteasome cylinder, preventing unrestricted exposure of cytosolic proteins to proteolysis.

Almost 25 years ago, two more β-type proteasome subunits that are homologous to β1 and β5 were identified: proteasome subunit β1i (also known as PSMB9 and LMP2, low molecular weight protein 2) and proteasome subunit β5i (also known as PSMB8 and LMP7; Glynne et al., 1991; Kelly et al., 1991; Ortiz-Navarrete et al., 1991). These subunits are encoded by genes in the MHC class II region and are induced by IFN-γ and TNF-α (Aki et al., 1994), leading to the designation of these subunits as “immunosubunits” and the complex they form as the “immunoproteasome” (Tanaka, 1994; **Figure 2A**). A third IFN-γ-inducible proteasome subunit, this one outside of the MHC region, was subsequently identified: proteasome subunit β2i (also known as PSMB10, LMP10, and MECL-1, multicatalytic endopeptidase complex-like 1), which is homologous to β2 (Groettrup et al., 1996a; Hisamatsu et al., 1996; Nandi et al., 1996). Expression of the three immunosubunits following IFN-γ stimulation is mediated by interferon (IFN) regulatory factor-1 (IRF-1; Chatterjee-Kishore et al., 1998; Foss and Prydz, 1999; Brucet et al., 2004; Namiki et al., 2005). Type I IFNs can also upregulate the immunoproteasome, although higher concentrations are needed to achieve the same upregulation induced by IFN-γ (Shin et al., 2006; Freudenburg et al., 2013a,b).

An additional type of specialized proteasome, termed the thymoproteasome, was identified in cortical thymic epithelial cells (cTECs). This proteasome contains the immunosubunits β1i and β2i as well as a cTEC-specific proteasome subunit β5t (also known as PSMB11; **Figure 2B**). Expression of β5t is essential for positive selection of T cells, and expression of the homologous subunits β5 or β5i cannot compensate for deficiency in this specialized subunit (Murata et al., 2007; Nitta et al., 2010; Xing et al., 2013).

## IMMUNOPROTEASOME FORMATION AND TISSUE EXPRESSION

Immunoproteasome assembly occurs in a cooperative manner whereby the immunosubunits interact with one another to favor the assembly of immunoproteasomes containing all three immunosubunits. This occurs even in cells that coexpress both standard and immunosubunits (Griffin et al., 1998). The immunosubunit β1i is incorporated more quickly than β1, and incorporation of β2i depends on that of β1i (Groettrup et al., 1997; De et al., 2003). Incorporation of β5i is required for the maturation (removal of the propeptides) of β1i and β2i, which would otherwise prevent their catalytic activity (Groettrup et al., 1997; Griffin et al., 1998). β5i is the only subunit that can be incorporated into immunoproteasomes independently of the other subunits, allowing for the existence of “mixed” or intermediate proteasomes that contain both β1i/β5i or β5i without other immunosubunits (Griffin et al., 1998; Kingsbury et al., 2000). Mixed proteasomes are present in some human tissue types in the absence of stimulation or inflammation, especially the liver and colon, but not the heart. They are particularly abundant (50% or greater of the proteasomes in cell lysates) in APCs, such as monocytes and both immature and mature DCs (Guillaume et al., 2010).

The spleen has the highest level of baseline immunoproteasome expression and activity compared to other organs (Noda et al., 2000; Ebstein et al., 2012). This makes sense given that the immunoproteasome is abundantly expressed in cells of hematopoietic origin, including professional APCs such as macrophages and B cells, found in the spleen (Frisan et al., 2000; Haorah et al., 2004). Malignant cell lines derived from B cells or multiple myeloma express high levels of immunoproteasome subunits (Frisan et al., 1998; Altun et al., 2005). There has been some disagreement regarding the regulation of immunoproteasome expression in DCs. Initial reports suggested that immature DCs constitutively express immunoproteasomes at equal levels to that of the standard proteasome. Upon maturation, immunosubunit expression is dramatically upregulated and synthesis of new proteasomes switches exclusively to immunoproteasomes (Macagno et al., 1999). Later reports showed that immunoproteasome content is unchanged or even decreased in DCs following maturation (Li et al., 2001; Macagno et al., 2001; Ossendorp et al., 2005). The disagreements regarding immunoproteasome expression in DCs may have been due to lack of immunosubunit-specific antibodies at the time of those studies. A more recent study demonstrated the presence of mostly immunoproteasomes and mixed proteasomes (β1/β2/β5i and β1i/β2/β5i) in immature DCs that does not change after maturation (Guillaume et al., 2010).

Constitutive expression of immunoproteasome subunits by immune cells appears to be independent of external signaling requirements, such as persistent stimulation by cytokines *in vivo*, because immune cells maintain their immunoproteasome expression *in vitro* in the absence of cytokines or other external stimuli. Rather, the high basal levels of immunoproteasome expression in immune cells are likely due to permanent activation of intracellular signaling pathways. One report demonstrated minor reductions in β1i and β5i mRNA in thymus and spleen tissue of mice lacking either type I or type II IFN receptors (Lee et al., 1999), but a second study demonstrated that the spleens of IFN-γ-deficient mice have levels of immunoproteasome protein expression similar to that of wild-type mice (Barton et al., 2002). In spleens of STAT1^-/-^ mice, however, mRNA and protein expression of immunoproteasome subunits is markedly reduced (Lee et al., 1999; Barton et al., 2002), suggesting that basal immunoproteasome expression does not require IFN-γ signaling (and therefore phosphorylated STAT1), but it is at least partially dependent on non-phosphorylated STAT1. This is supported by evidence that non-phosphorylated STAT1 and IRF1 form a complex that occupies the IFN-γ-activated sequence (GAS) elements of the β1i promoter to support its constitutive expression (Chatterjee-Kishore et al., 2000). There is still some basal immunoproteasome expression in spleens and thymus of STAT1^-/-^ mice. This may reflect equal reduction of immunoproteasome subunits in all immune cell types present in these tissues (i.e., STAT1 greatly enhances basal expression), or could be due to complete absence of immunoproteasome expression in some cell types and not others (i.e., a cell-type-specific dependence on STAT1 for basal expression).

While non-immune cells express standard proteasomes almost exclusively, immunoproteasome expression can be induced in such cells following exposure to IFN-γ. As mentioned above, type I IFNs can also upregulate the immunoproteasome, although less efficiently than IFN-γ (Shin et al., 2006; Freudenburg et al., 2013a,b). An initial report suggested that TNF-α could act synergistically with IFN-γ to upregulate β5i expression (Hallermalm et al., 2001), implying that other proinflammatory cytokines may be capable of regulating immunoproteasome expression. However, in three murine cell lines of non-hematopoietic origin, only IFN-γ was capable of upregulating immunoproteasome subunit expression, and there was no effect of IL-1, IL-4, IL-6, TNF-α, TGF-β, IL-3, or GM-CSF treatment on immunoproteasome subunit protein levels (Barton et al., 2002). Therefore, it seems that IFN signaling is required for immunoproteasome induction in non-hematopoietic cells and that other proinflammatory cytokines cannot regulate immunoproteasome expression. There are some exceptions to reports that non-immune cells express exclusively standard proteasomes. For instance, constitutive immunoproteasome expression has been reported in immune-privileged sites that are highly unlikely to be subject to persistent cytokine stimulation, such as the eye and brain (Singh et al., 2002; Piccinini et al., 2003; Ferrington et al., 2008), suggesting a role for immunoproteasomes in non-immune processes.

## 26S AND 20S PROTEASOMES

**FIGURE 3:**
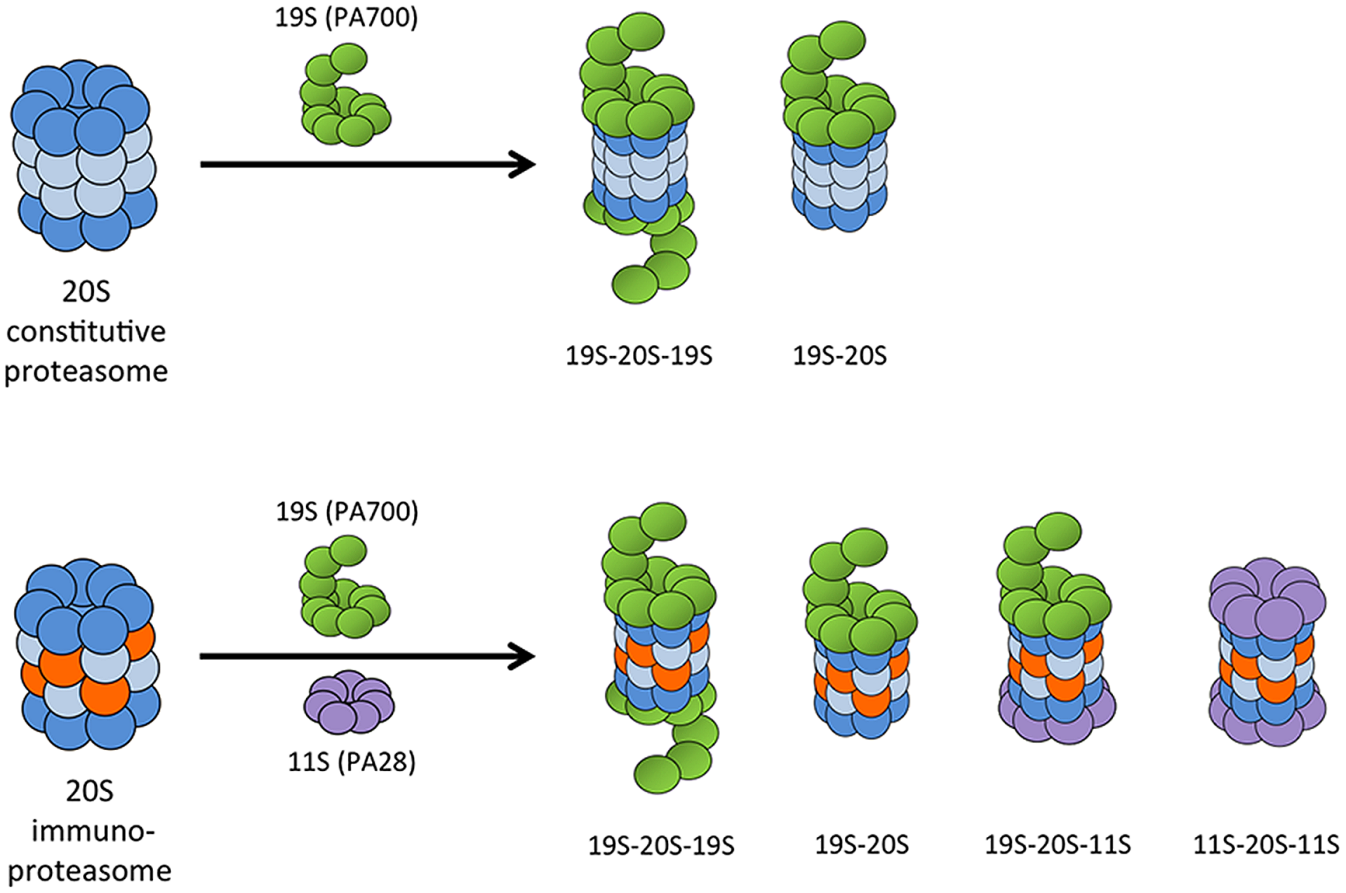
**Possible combinations of 20S proteasome core with proteasome activator complexes.** The 19S (PA700) regulatory cap can associate at one or both ends of the 20S proteasome core to form an asymmetric 26S proteasome or a 26S proteasome, respectively. The IFN-γ-induced 11S (PA28) regulatory complex can bind at the free end of a 19S-20S complex to form a hybrid proteasome, or it can associate with both ends of the 20S immunoproteasome core.

Proteasomes exist in many forms in cells, with different regulatory or activator cap complexes that associate with the 20S core to control access to the proteolytic inner chamber (Baumeister et al., 1998). The α rings serve as scaffolds for the β subunits during proteasome assembly, but they also serve as binding sites for regulatory and activator complexes. The 26S proteasome, which is composed of a 20S core particle and one or two 19S (also known as PA700) regulatory caps, degrades proteins in an ATP- and largely polyubiquitin-dependent manner. The 19S regulator complex recognizes and binds polyubiquitin moieties, then unfolds and feeds substrate proteins into the 20S core for degradation (Navon and Goldberg, 2001). Although 26S proteasomes preferentially degrade ubiquitinated proteins, degradation can occur without ubiquitination if the protein is first denatured (Benaroudj et al., 2001). The 26S proteasome is responsible for the majority of normal protein turnover within cells. Because the α subunits are unchanged between different types of proteasomes, the 19S regulator complex can associate with 20S cores containing either standard or immunosubunits. This makes possible a number of different proteasome and immunoproteasome combinations: 20S alone, asymmetric 26S proteasomes (19S-20S), or 26S (19S-20S-19S), with each capable of having either the standard or immunosubunits in the 20S core (**Figure 3**).

It was originally thought that cells have little to no free 20S proteasome, and that the 20S complex is incapable of acting independently of its regulators or activators (Rivett et al., 2001). Without the 19S regulatory cap, the 20S proteasome does not have the ability to recognize and unfold polyubiquitinated proteins. The 20S core seems to exist in an autoinhibited state, where the N-terminal tails of the α subunits at the openings on either end of the complex prevent substrate access to the internal proteolytic chamber (Groll et al., 2000). Binding of activator or regulatory complexes to the 20S core displaces the N-terminal tails, opening a channel into the lumen of the proteasome (Whitby et al., 2000; Köhler et al., 2001). However, even in the absence of activating agents [such as heating or low detergent concentrations (Coux et al., 1996)], the 20S core is capable of degrading proteins at low, albeit detectable and reproducible, rates. Degradation of proteins by the 20S core probably involves partial or transient opening of the inner channel and is not an active process (Osmulski and Gaczynska, 2000, 2002; Köhler et al., 2001; Osmulski et al., 2009). Indeed, the 26S proteasome and immunoproteasome hydrolyze unstructured polypeptides at rates nearly 10-fold higher compared to 20S proteasomes and immunoproteasomes (Benaroudj et al., 2001; Cascio et al., 2001; Raule et al., 2014a). However, the free 20S core is capable of binding to and degrading proteins in a process that is both ATP- and ubiquitin-independent (Ciechanover, 1994; Coux et al., 1996). Rather than recognizing ubiquitin moieties, the 20S proteasome has a selective preference for degradation of damaged or oxidized proteins, while the 26S proteasome does not (Reinheckel et al., 1998; Davies, 2001; Pickering et al., 2010). Thus, it appears that the majority of normal protein turnover occurs through the 26S proteasome, while the 20S proteasome plays a specialized role in degradation of damaged or oxidized proteins. It has been suggested that oxidation may act as a marker for targeting proteins to the MHC class I pathway (Teoh and Davies, 2004). This notion, termed the PrOxI (protein oxidation and immunoproteasome) hypothesis, would represent a new mechanism of substrate generation by the proteasome and may act in concert with other pathways (such as the DRiPs pathway discussed below) to efficiently generate peptides for MHC class I presentation.

## PROTEASOME PROCESSING OF PEPTIDES FOR MHC CLASS I

The changes in proteasome subunit composition from standard to immunosubunits in response to IFN-γ stimulation alter the proteolytic activity of the complex. Purified 20S and 26S immunoproteasomes from IFN-γ-treated cells substantially increase the rate at which they cleave after hydrophobic and basic residues (Driscoll et al., 1993; Gaczynska et al., 1993; Aki et al., 1994) and decrease the rate of cleavage after acidic residues (Gaczynska et al., 1996). As the vast majority of peptides presented on MHC class I have hydrophobic or basic C-termini, the immunoproteasome is thought to generate peptides better suited to binding to MHC class I molecules compared to the constitutive proteasome and thus be more efficient at eliciting immune responses (Driscoll et al., 1993; Früh et al., 1994). Using ovalbumin (OVA) as a protein substrate, rates of degradation by 26S proteasomes and immunoproteasomes, or 20S proteasomes and immunoproteasomes, are indistinguishable (Cascio et al., 2001). All produce peptides of similar sizes ranging between 3 and 22 residues. 26S particles yield peptides with a mean size of 7–8 residues, while the mean size of products from 20S particles is slightly larger, at 8–9 residues (Kisselev et al., 1999). The different β subunits therefore do not affect rates of protein degradation or peptide size, but rather seem to affect the cleavage sites within a protein to generate peptides with more hydrophobic C-termini.

It was uncertain whether proteasomes cleave proteins to the exact length (8–10 residues) that would be directly loaded onto MHC class I molecules, or whether they produce larger precursors that are further cleaved by other peptidases. Some initial experiments indicated that isolated 20S proteasomes could cleave larger peptides to antigenic epitopes (Niedermann et al., 1995, 1999; Lucchiari-Hartz et al., 2000). However, these experiments used short (less than 50 amino acid) precursors that are likely very different from the ubiquitinated or damaged full-length protein substrates that the proteasome would encounter under normal conditions. 20S proteasomes also release a different spectrum of products than do 26S proteasomes (Kisselev et al., 1999; Emmerich et al., 2000). A number of studies have indicated that proteasomes release N-extended versions of antigenic peptides, which are then trimmed by aminopeptidases in the cytosol (Stoltze et al., 2000) or ER (Snyder et al., 1994; Craiu et al., 1997a). Moreover, one of these aminopeptidases, leucine aminopeptidase (LAP), is induced by IFN-γ (Beninga et al., 1998). Following IFN-γ treatment, cytosolic LAP activity accounted for all trimming of an N-terminal extended version of the well-studied OVA-derived epitope SIINFEKL to the correct length.

Although immunoproteasomes degrade proteins at the same rate as standard proteasomes, immunoproteasomes generate more antigenic peptides than standard proteasomes (Cascio et al., 2001). Degradation of OVA by standard 26S proteasomes isolated from muscle tissue produces SIINFEKL or an N-extended version only 6–8% of the time. When 26S immunoproteasomes from the spleen are used, the percentage of peptides containing SIINFEKL at the C-terminus increases to 11%. This is not due to an increase in the amount of final SIINFEKL peptide generated, as standard proteasomes and immunoproteasomes release the same amount of SIINFEKL. Instead, 20S or 26S immunoproteasomes generate 2–4 times the amount of N-extended versions of this peptide, which could be trimmed by the cytosolic enzyme LAP, compared to their standard proteasome counterparts. Therefore, it seems that the effect of IFN-γ on antigenic peptide generation within cells is at least threefold: changes from standard to immunosubunits in the 20S proteasome core directly affect C-terminal processing and generate more N-extended versions of antigenic peptides, while induction of aminopeptidase activity in the cytosol alters N-terminal processing.

## IFN-γ-INDUCED PROTEASOME REGULATOR PA28

Another protein complex induced by IFN-γ is PA28 (also known as REG or 11S), a large regulatory complex that binds the ends of the 20S proteasome in an ATP-independent manner (**Figure 3**). In mammals, PA28 is made of two homologous subunits, PA28α (REGα or PSME1) and PA28β (REGβ or PSME2; Honoré et al., 1993; Realini et al., 1994; Ahn et al., 1995, 1996; Jiang and Monaco, 1997; Tanahashi et al., 1997; Rechsteiner et al., 2000). A third PA28 family member, PA28γ (also known as REGγ or Ki antigen), is structurally related to PA28α and PA28β. PA28γ associates with 20S proteasomes primarily in the nucleus, and unlike PA28α/β, it is not induced by IFN-γ (Tanahashi et al., 1997). PA28γ does not appear to play a role in the immune response, but is involved in regulation of cell proliferation and tumorigenesis through multiple pathways (He et al., 2012; Ali et al., 2013; Li et al., 2013; Levy-Barda et al., 2014). It was originally predicted that PA28α/β either enhanced the rate of protein degradation by proteasomes or generated peptides better suited to binding to MHC class I. However, the biological functions of PA28 are still relatively unknown. PA28 does not enhance rates of protein degradation by either the standard proteasome or the immunoproteasome. In fact, PA28-20S particles degrade proteins at the same slow rate as 20S particles alone (Raule et al., 2014a). PA28 appears to enhance the ability of the 20S proteasome to degrade short peptide substrates, but not proteins or polyubiquitinated proteins (Dubiel et al., 1992; Ma et al., 1992). PA28 is also able to associate with asymmetric 26S proteasomes (20S proteasomes with only one 19S regulatory complex, usually denoted as 19S-20S) to form hybrid proteasomes (19S-20S-PA28; **Figure 3**; Hendil et al., 1998; Tanahashi et al., 2000; Kopp et al., 2001; Cascio et al., 2002). Hybrid proteasomes hydrolyze 3- and 4-residue peptides at faster rates than standard 26S particles.

An extensive study of the molecular mechanisms of PA28 was recently undertaken by Raule et al. (2014a) who performed *in vitro* degradation of full-length proteins (insulin-like growth factor-1 and casein) by 20S, 26S, and PA28α/β-20S immunoproteasomes and analyzed the range of peptides released. Rather than increase the fraction of 8–10 residue peptides that is generated, association of PA28 with 20S immunoproteasomes reduces it from 10% to approximately 6% of the total, with the majority of peptides being <6 amino acids in length. This may occur through allosteric modification of proteasome active sites by PA28α/β. Alternatively, PA28α/β may control the eﬄux of longer peptides out of the proteolytic chamber and contribute to their ongoing hydrolysis (Raule et al., 2014a; Yang and Schmidt, 2014).

Binding of PA28 to the 20S catalytic core also appears to favor the release of a specific subset of longer peptides with an acidic C terminus, several of which contain the correct C-terminal anchor residue required for binding to MHC class I (Raule et al., 2014a). Several studies have demonstrated that PA28 expression enhances MHC class I presentation of certain antigens (Groettrup et al., 1996b; Schwarz et al., 2000; van Hall et al., 2000; Sun et al., 2002) but not others (Murata et al., 2001). It was proposed that this small fraction of peptides specifically generated by PA28-20S immunoproteasomes may be important in stimulating an effective CD8 T cell response under certain pathophysiological conditions in which a ubiquitin-independent proteolytic pathway is favored. However, since the vast majority of peptides released by PA28-20S immunoproteasomes are too short to serve as MHC class I antigens, an alternative possibility is that PA28 may play a regulatory role by preventing excessive cytotoxic response against self-antigens, and decrease the risk of autoimmune reactions. A recent study demonstrated that purified PA28α/β increases the capacity of both the constitutive 20S proteasome and the immunoproteasome to selectively degrade oxidized proteins in response to hydrogen peroxide-induced oxidative stress, supporting a role for PA28 that is independent of MHC class I antigen processing (Pickering and Davies, 2012).

Although PA28 does not stimulate proteolytic degradation under normal conditions, PA28 does increase catalytic rates of the immunoproteasome under conditions of ATP depletion (Freudenburg et al., 2013b). The implications of PA28 regulation by cellular ATP levels are unknown. Proinflammatory cytokines, such as IL-1 and IFNs, significantly decrease total cellular ATP levels (Corbett et al., 1992; Collier et al., 2006). It is possible that decreases in ATP levels that could occur during inflammatory conditions such as viral infection trigger increased association of PA28 with 20S immunoproteasomes and enhance rates of protein degradation. However, given that the majority of peptides degraded by PA28-20S proteasomes and immunoproteasomes are not suitable for binding to MHC class I, it seems unlikely that this added level of regulation is related to MHC class I antigen processing. It may instead be related to possible roles for PA28 in degradation of oxidized proteins or decreasing the potential for autoimmune reactions at sites of inflammation, as discussed above. If PA28 does dampen autoimmunity, then one would expect to see an increase in autoimmune responses in PA28-deficient mice after an inflammatory response. These are intriguing possibilities that bear further investigation.

## STRATEGIES TO STUDY IMMUNOPROTEASOME FUNCTION

Until recently, traditional gene deletion has been the main strategy employed to study immunoproteasome function. There are numerous drawbacks to this approach. Due to cooperative assembly of immunoproteasomes, deficiency in one subunit could affect the structure and assembly of the 20S core, as well as impair binding or activity of regulatory subunits. Mice deficient in one or more of the immunosubunits since birth could develop compensatory mechanisms of proteasome or immunoproteasome assembly, leading to alteration in subunit composition that could detrimentally affect peptide processing. They could also have defects in maturation of the immune system, since the thymoproteasome (composed of β1i, β2i, and the cTEC-specific subunit β5t) is important for positive selection of T cells (Murata et al., 2007). Most studies of immunoproteasome function have been undertaken with mice in which only one or two immunosubunits are deleted, rather than all three. It is possible that a standard proteasome subunit is able to compensate when only one or two immunosubunits are missing, in which case a phenotype would not be observed unless the mice are lacking all immunoproteasome activity. Therefore, caution must be taken in drawing conclusions from studies using mice deficient in one or more immunosubunits.

Small molecule peptide screens have led to the identification of inhibitors specific to immunoproteasome activity. The use of small molecule inhibitors offers several advantages over traditional gene deletion approaches, the most obvious of which is their potential for use as therapeutics. Because small molecule inhibitors are unlikely to affect the assembly or structure of the immunoproteasome, they allow for the study of how the catalytic activity of a specific subunit affects immune responses. Furthermore, these inhibitors are unlikely to affect the positive selection of T cells in the thymus, since most studies are undertaken in adult mice after maturation of the immune system.

The first reported immunoproteasome-specific inhibitor, PR-957 (now known as ONX 0914), inhibits β5i with an IC_50_ value of approximately 10 nM (Muchamuel et al., 2009). ONX 0914 is 20- to 40-fold more selective for β5i than for the next two most sensitive subunits, β1i and β5. A newer β5i-specific inhibitor, PR-924, specifically targets β5i and has less specificity toward other subunits compared to ONX 0914 (Parlati et al., 2009). UK-101 was the first identified compound to specifically inhibit β1i (Ho et al., 2007; Wehenkel et al., 2012), with two more (IPSI-001 and YU-102) identified shortly thereafter (Kuhn et al., 2009; Miller et al., 2013). Leupeptin is a recently described inhibitor of the trypsin-like activity of the proteasome (β2 and β2i) that does not affect activity of other β subunits (Kisselev et al., 2006; Raule et al., 2014b). There are currently no available compounds that specifically inhibit the activity of β2i. A recent crystal structure of the murine constitutive proteasome and the immunoproteasome in complex with ONX 0914 revealed important structural differences in the binding pockets of the different subunits (Huber et al., 2012). While the crystal structures demonstrated that β1 and β5 have distinct substrate binding pockets that are distinct from those of their immunosubunit counterparts, the substrate binding pockets of β2 and β2i are essentially identical. Therefore, it will be difficult to develop β2i inhibitors that do not also target its constitutive counterpart. Several other proteasome- and immunoproteasome- specific inhibitors are in development and are of significant interest as potential therapeutic agents (Miller et al., 2013).

## IMMUNOPROTEASOME AND ACTIVATION OF THE NF-κB PATHWAY

The nuclear factor-κB (NF-κB/Rel) family of transcription factors plays a central role in regulation of immunity and inflammation. NF-κB transcription factors interact as homodimers or heterodimers with other NF-κB family members, including p65 (RelA), RelB, c-Rel, p50 (NF-κB1), and p52 (NF-κB2). Under normal conditions, these factors exist in the cytoplasm in an inactive state because of interaction with inhibitory IκB proteins (IκBα, IκBβ, IκBε) or the unprocessed forms of NF-κB1 and NF-κB2 (p105 and p100, respectively). The NF-κB pathway is activated in response to many different stimuli, including exposure to inflammatory cytokines such as TNF-α or IL-1 family members (Vallabhapurapu and Karin, 2009). In the canonical or classical pathway of NF-κB activation, the proteasome degrades IκBα, releasing the active NF-κB dimer (usually p65/p50) and allowing translocation to the nucleus. In the non-canonical or alternative pathway of NF-κB activation, the proteasome degrades the inhibitory portion of p105 or p100 to generate the active transcription factors p50 or p52. These transcription factors can then associate with p65, RelB, or each other to form homodimers and heterodimers. The classical and alternative NF-κB pathways regulate distinct sets of target genes, in part because different populations of NF-κB dimers are regulated by either IκBα degradation or p100 processing (Hayden and Ghosh, 2008).

It is widely accepted that the standard proteasome plays a crucial role in the processing of the p105 precursor of the p50 subunit and in the degradation of IκBα (Palombella et al., 1994; Traenckner et al., 1994). However, a role for the immunoproteasome in NF-κB pathway activation is controversial. Non-obese diabetic (NOD) mice were reported to have a specific defect in β1i production that resulted in defective activation of NF-κB (Hayashi and Faustman, 1999). This finding has been debated, but contradictory results were likely due to different cell populations and the phenotype (non-diseased versus diseased) of NOD mice analyzed (Hayashi and Faustman, 1999; Kessler et al., 2000; Runnels et al., 2000). Nevertheless, Hayashi and Faustman (1999) did directly demonstrate impaired NF-κB activation in lymphocytes from β1i^-/-^ mice. The T2 human lymphocyte cell line, which lacks both β1i and β5i, has substantial defects in NF-κB activation compared to the parental T1 cell line and is sensitive to TNF-α-induced apoptosis (Hayashi and Faustman, 2000).

In support of a role for the immunoproteasome in NF-κB activation, another study reported delayed termination of the classical NF-κB activation pathway and reduced activation of transcription factors associated with the alternative NF-κB pathway in β1i^-/-^ mice, but not β1i/β5i double knockout mice (Maldonado et al., 2013). B cells isolated from β1i^-/-^ mice exhibit slightly delayed IκB degradation, although the authors posited that defects in these mice were likely due to the presence of mixed proteasomes containing β1, β2i, and β5i because a B cell phenotype was not observed in mice lacking both β1i and β2i (Hensley et al., 2010). It is important to note that the mixed proteasomes may have abnormal function that is directly responsible for observed defects in NF-κB activation in β1i^-/-^ mice (discussed in more detail below). If that is the case, then the deficiencies in these cell types in β1i^-/-^ mice are not a true reflection of deficient immunoproteasome function.

In support of this possibility, another study used two small molecule inhibitors of the immunoproteasome, UK-101 and LKS01, which target β1i and β5i, respectively, to study the role of the immunoproteasome in NF-κB activation in lung and pancreatic adenocarcinoma cells (Jang et al., 2012). Their results suggest that the catalytic activity of β1i and β5i is not required for canonical NF-κB activation (as measured by IκB degradation), and they support the notion that deficiencies in NF-κB activation in β1i^-/-^ mice may instead be an artifact of mixed proteasomes. One study demonstrated reduced NF-κB activation in cardiomyocytes and B-cell-depleted splenocytes in β5i^-/-^ mice following exposure to IFN-γ (Opitz et al., 2011). However, because NF-κB activation in this study was measured by assessing p50 levels in whole cell homogenates, it is unknown whether the reduced levels in β5i^-/-^ mice were due to impaired activation of the classical or alternative NF-κB pathway.

Since some studies have reported impaired activation of the alternative NF-κB pathway in β1i^-/-^ mice, it will be important to repeat the UK-101 and LKS01 inhibitor studies to determine whether the catalytic activity of β1i or β5i is important in the alternative pathway of NF-κB activation by measuring p100 or p105 degradation. Additionally, it remains to be determined whether other cell types, such as those of the immune system that express the immunoproteasome constitutively, use immunoproteasome activity in the either the classical or alternative pathway of NF-κB activation.

## IMMUNOPROTEASOME FUNCTIONS IN ANTIGEN PROCESSING AND VIRAL INFECTION

Immunoproteasome function appears to be important for a variety of host responses to viral infection, although the specific effects depend on the virus studied and the models used (summarized in **Table 1**). Because β1i and β5i are encoded on the MHC locus, it was originally thought that the major function of the immunoproteasome is to regulate the immune response via optimization of MHC class I peptide processing. Although proteasome activity in general is required for MHC class I antigen presentation, the immunoproteasome does not appear to be essential for that function. In fact, some epitopes are processed more efficiently by the 20S proteasome than the immunoproteasome [(Morel et al., 2000; Van den Eynde and Morel, 2001) and discussed above]. However, the immunoproteasome is certainly more effective than the standard proteasome at producing many MHC class I epitopes, particularly immunodominant epitopes derived from infectious organisms. Many of the epitopes processed inefficiently by the immunoproteasome are derived from self proteins (Van den Eynde and Morel, 2001). While these epitopes may be important for generating an immune response to tumor antigens and could have implications for design of cancer vaccines, it is unlikely that they play a role in the immune response to an infectious organism.

**Table 1:** Contributions of immunoproteasome function during viral infection.

Virus	Immunoproteasome inhibition/deficiency	Effects on CD8 T cell function	Other effects on immune response and inflammation
MCMV	β5i^-/-^	• Nearly all MCMV-specific epitopes decreased • Memory-inflating epitopes less dependent on the immunoproteasome	
HBV	β1i^-/-^β5i^-/-^	• Moderately reduced CD8 T cell responses to HBV polymerase and envelope proteins	
Influenza	β1i^-/-^	• Reduced capacity of APCs to generate influenza NP epitope • Decreased responses to immunodominant epitopes • Reduction in overall number of influenza virus-specific CD8 T cells	• B cells display survival defect and reduced isotype switching • DCs show reduced innate cytokine production • Influenza virus titers in sera reduced
LCMV	β2i^-/-^	• Reduced response to some LCMV-derived epitopes	
	β1i^-/-^β2i^-/-^ plus ONX 0914	• Largely normal CD8 T cell responses	
	β5i^-/-^		• Delayed and less robust CNS inflammation
	β1i^-/-^β2i^-/-^β5i^-/-^	• Significantly decreased CD8 T cell responses	• MHC class II presentation and CD4 T cell responses unchanged
CVB3	β5i^-/-^	• No effect on CD8 T cell responses in the heart	• No effect on CVB3 replication • Increased severity of myocardial tissue damage • Increased polyUb conjugates and oxidant-damaged proteins in the heart • Increased apoptosis in the heart

The immunoproteasome appears to facilitate T cell responses that are independent of MHC class I antigen presentation. A common phenotype of immunoproteasome-deficient mice is reduced number of CD8 T cells in the spleen, supporting contributions of the immunoproteasome to T cell development or maturation (Van Kaer et al., 1994; Hensley et al., 2010; Basler et al., 2011). A number of studies have reported increased CD4/CD8 T cell ratios β2i^-/-^ mice (Chen et al., 2001; Caudill et al., 2006; Basler et al., 2013a), and this has recently been ascribed to a T-cell-intrinsic process that occurs independently of both thymic selection and antigen processing (Zaiss et al., 2008). T cells from β1i^-/-^, β2i^-/-^, or β5i^-/-^ mice are impaired in proliferation and survival when transferred into virus-infected wild-type mice, suggesting a role for the immunoproteasome in the expansion and maintenance of T cell populations during an immune response (Chen et al., 2001; Basler et al., 2006; Moebius et al., 2010).

The immunoproteasome may also play a critical role in B cell development, as mice deficient in β1i, but not β1i/β2i or β5i/β2i, have reduced numbers of mature B cells in the spleen (Hensley et al., 2010). These authors reported reduced survival and impaired immunoglobulin (Ig) isotype switching in B cells from β1i^-/-^ mice compared to wild-type B cells. A separate report was unable to recapitulate the finding of reduced B cells in β1i^-/-^ mice, and it demonstrated equivalent numbers of CD19^+^ B cells in the spleens of mice deficient in β1i, β2i, or both β1i/β2i (Basler et al., 2011). B cell responses were not examined in detail in that study. Therefore, the role of the immunoproteasome in B cell development or induction of a humoral response following a viral infection is still largely undefined.

The immunoproteasome, or at least the β5i subunit, plays a critical role in generating nearly all mouse cytomegalovirus (MCMV)-derived epitopes (Hutchinson et al., 2011). Interestingly, memory “inflating” epitopes, or epitopes for which the pool of specific CD8 T cells is sustained or even increased over time, show a reduced dependence on the immunoproteasome compared to non-inflating epitopes. This suggests that immunoproteasomes play a role in stimulating immune responses during acute infection, but not during chronic MCMV infection. Although this study did not monitor the effect of β5i deficiency on MCMV viral loads over time, it was suggested that β5i deficiency likely would not have an impact on MCMV replication because neither CD8 nor MHC class I deficiency have an impact on viral loads in this model.

The immunoproteasome (subunits β1i and β5i) moderately influences the magnitude and specificity of CD8 T cell responses to hepatitis B virus (HBV) polymerase and envelope proteins (Robek et al., 2007). Although type I IFNs and IFN-γ inhibit HBV replication, the antiviral effect of IFNs occurs independently of their induction of β1i and β5i.

The majority of studies examining the effect of immunoproteasome deficiency on the generation of antigenic epitopes during viral infection have been performed with influenza virus or LCMV, two well-studied viruses for which the immunodominant CD8 T cell epitopes are known. The WE strain has been the most commonly used strain for LCMV-immunoproteasome studies, while the Armstrong and clone 13 strains have been used far less frequently. It is important to consider potential differences associated with the use of different LCMV strains, which vary greatly in terms of their interactions with host immune function and their ability to persist in an infected host.

APCs from β1i^-/-^ mice show a reduced capacity to generate an influenza virus nucleoprotein-specific epitope, while presentation of OVA-derived antigens was unaffected (Van Kaer et al., 1994). Two later studies using seven defined peptides from influenza virus showed that β1i (and to a lesser extent the other immunoproteasome subunits) plays a major role in establishing the immunodominance hierarchy of responding CD8 T cells (Chen et al., 2001; Pang et al., 2006). Responses to the two most immunodominant epitopes significantly decreased in β1i^-/-^ mice. One of these was due to decreased generation of the epitope by APCs, while the other was due to reduced frequency of epitope-specific T cells in the CD8 T cell repertoire. The overall number of influenza virus-specific CD8 T cells was decreased in β1i^-/-^ mice, even when β1i^-/-^ CD8 T cells were restimulated with APCS (influenza virus-infected splenocytes) from wild-type mice. Because this defect was observed for epitopes produced equally by standard proteasomes and immunoproteasomes, it was suggested that immunoproteasomes might play a role in T cell activation and proliferation.

Interestingly, influenza virus titers are reduced approximately 50% in sera of β1i^-/-^ mice. While B cells from influenza virus-infected β1i^-/-^ mice proliferate as well as those from wild-type mice, they display a survival defect and impaired Ig isotype switching. DCs from the same mice show reduced innate cytokine production in response to influenza virus infection (Hensley et al., 2010). The altered response of many cell types in β1i^-/-^ mice to influenza virus is likely due to the presence of mixed proteasomes containing β2i and β5i.

Care must be taken in the interpretation of results obtained using β1i^-/-^ mice, since results cannot be attributed solely to absence of β1i catalytic activity. Instead, any effect may be due to dysregulated proteasome assembly and function. While mixed proteasomes containing both standard and immunosubunits have recently been isolated from wild-type mice, these mixed proteasomes contain either β1i/β5i or just β5i, in accordance with the rules of cooperative immunoproteasome assembly (Guillaume et al., 2010). The authors of that study were unable to detect the presence of mixed proteasomes containing β2i, as all of the β2i subunits were associated with immunoproteasomes. Cooperative assembly rules should preclude formation of mixed proteasomes containing β2i, because both β1i and β5i are required for its inclusion in the immunoproteasome. It is possible that β5i could compensate (perhaps partially or inefficiently) for β1i in the assembly process, or that β2i could interact with the standard β1 subunit in the complete absence of β1i (as in β1i^-/-^ mice). This may explain the seemingly contradictory presence of mixed β2i/β5i proteasomes in β1i^-/-^ mice. It is doubtful that mixed proteasomes containing β2i exist in wild-type mice, although this has not been formally analyzed in all tissues or cell types. Mixed proteasomes (containing β1i/β5i or β5i alone) are highly expressed in human immature and mature DCs. Human monocytes also contain a particularly high abundance of mixed proteasomes, up to 50% of the total proteasome content. The mixed proteasome content of B and T cells is unknown. However, the finding that mixed proteasomes are expressed at high levels in some cell types, particularly APCs, suggests that they may play an important role in shaping CD8 T cell responses. Indeed, work by Zanker et al. (2013) using mice deficient in β1i, β2i, or β2i/β5i demonstrated that mixed proteasomes increase viral peptide diversity and broaden antiviral CD8 T cell responses to influenza virus.

Mice deficient in β2i have ~20% fewer CD8 T cells in the spleen and reduced response to some LCMV-derived epitopes (WE strain). This is not due to impaired generation or presentation of these epitopes, but rather to either decreased precursor frequency or reduced expansion of the epitope-specific T cells, further supporting a role for the immunoproteasome in T cell survival or expansion rather than just antigen presentation (Basler et al., 2006). One strategy that has been employed to study mice lacking all immunoproteasome activity has been to use β1i/β2i double knockout mice treated with the β5i-specific inhibitor ONX 0914 (Basler et al., 2011). Although these mice have fewer CD8 T cells in the spleen, and CD8 T cell responses to several LCMV-specific MHC class I epitopes are changed (two are increased and others are decreased; again WE strain), these double knockout mice otherwise mount largely normal CD8 T cell responses to LCMV infection. Spleen LCMV titers at 4 dpi were unchanged in immunoproteasome-deficient mice treated with ONX 0914, although it remains to be seen whether viral titers at later times (such as at 8 dpi, when CD8 T cell responses were analyzed) would be affected by lack of immunoproteasome activity. Splenocytes isolated from β1i^-/-^β2i^-/-^ ONX 0914-treated mice and stimulated with LPS or α-CD3/CD28 had reduced production of IL-6, TNF-α, and IFN-γ. However, this defect was observed in stimulated splenocytes from wild-type mice treated with ONX 0914 alone, suggesting a specialized function of β5i in promoting cytokine production that is not shared by the other immunosubunits. Because the cytokine studies in mice lacking immunoproteasome activity were performed in splenocytes stimulated *ex vivo* or in other models, it is unknown whether these mice display defects in cytokine production in response to LCMV or other viruses *in vivo.* The relatively modest effect of impaired immunoproteasome activity on the generation of LCMV-specific IFN-γ^+^ CD8 T cells suggests that overall IFN-γ production may be unaffected. However, β1i^-/-^β2i^-/-^ ONX 0914-treated mice may still have defects in production of other cytokines, such as IL-6 or TNF-α, in response to LCMV or other viruses.

Immunoproteasome subunits are transcriptionally induced in the brain following LCMV (WE strain) infection (Kremer et al., 2010). Mature immunoproteasome assembly is almost exclusively restricted to microglial-like cells, while only immunoproteasome precursors exist in astrocytes and do not exist at all in neurons or oligodendrocytes. LCMV-induced meningitis is delayed and less severe in β5i^-/-^ mice, suggesting a role for microglial immunoproteasomes in exacerbating immunopathology. The lack of mature immunoproteasome assembly in astrocytes may be due to a posttranslational mechanism that prevents excess immunoproteasome assembly in the brain. Since cells in the CNS regenerate poorly or not at all, inhibition of immunoproteasome assembly might be a strategy to protect these cells from immunopathological destruction.

The above studies demonstrate subtle and possible organ- or virus-specific roles for the immunoproteasome during viral infection using mice deficient in only one or two immunosubunits. To assess the role of complete immunoproteasome deficiency, a recent study generated mice deficient in all three immunoproteasome subunits (triply deficient mice; Kincaid et al., 2012). This had not been performed previously because the *LMP2* and *LMP7* genes (encoding β1i and β5i, respectively) are closely linked on the same chromosome and flank the *TAP1* transporter gene, so that breeding β1i^-/-^ and β5i^-/-^ mice with each other would not likely result in a double knockout but leave *TAP1* unaffected. However, Kincaid et al. (2012) used a sequential deletion strategy to first generate β1i/β5i doubly deficient mice, which were then bred to β2i^-/-^ mice to generate the triply deficient mice. APCs from these mice display profound defects in MHC class I antigen presentation, defects that are much more severe than those previously described in β1i, β2i, or β5i single knockout mice. These findings suggest that there may be functional overlap between the immunosubunits, and that the crucial role of immunoproteasomes in MHC class I antigen presentation has been obscured or underestimated by the use of mice deficient in only one immunosubunit. Triply deficient mice have an approximately 50% reduction in surface levels of MHC class I (Kincaid et al., 2012). This is likely due to a reduction in the supply of peptides available to bind to MHC class I molecules within the cell, rather than a defect in MHC class I expression itself. Of note, a similar 50% reduction of MHC class I surface expression is also observed in β5i^-/-^ mice, but not mice lacking either β1i or β2i, probably because β5i^-/-^ mice have a more severe defect in immunoproteasome assembly than β1i^-/-^ or β2i^-/-^ mice (Fehling et al., 1994). Presentation of nearly all MHC class I epitopes examined is significantly decreased in immunosubunit triply deficient mice both *in vitro* and *in vivo*. During LCMV infection (Armstrong strain), triply deficient mice display substantially weaker CD8 T cell responses than wild-type mice. This is due to defects in antigen presentation (and not to pleiotropic effects on T cells), because weaker T cell responses are also observed in wild-type T cells transferred into triply deficient mice. MHC class II epitope presentation and CD4^+^ T cell responses to LCMV are similar in wild-type and triply deficient mice, suggesting that the immunoproteasome does not affect processing of MHC class II antigens. It remains to be seen whether complete immunoproteasome deficiency (and the resulting substantially weaker CD8 T cell response) affects LCMV replication or other virus-induced inflammatory responses, such as cytokine production.

In addition to defects in antigen presentation, mice deficient in one or multiple immunoproteasome subunits have a peptide repertoire that substantially differs from wild-type mice, leading to rejection of wild-type cells when introduced into immunoproteasome-deficient mice (Toes et al., 2001; de Verteuil et al., 2010; Kincaid et al., 2012). The finding that standard proteasomes and immunoproteasomes generate such vastly different peptide repertoires has important implications. Under non-inflammatory conditions, the peptides presented on DCs (which constitutively express both standard proteasomes and immunoproteasomes) will be significantly different from the peptides displayed on non-immune parenchymal cells (which express only standard proteasomes). This implies that CD8 T cells stimulated by DCs may not efficiently recognize peptides displayed by non-immune cells until immunoproteasomes are induced in those non-immune cells by IFN. In cells that do not respond to IFN-γ and/or do not express immunoproteasomes, such as cells infected with a virus that inhibits IFN-γ signaling, this could suppress CD8 T cell responses and contribute to immune evasion. The differences in peptide repertoires produced by standard proteasomes and immunoproteasomes also have implications for acute inflammatory responses and vaccine design. During LCMV (WE strain) or *Listeria monocytogenes* infection, standard proteasomes in the liver are almost completely replaced by immunoproteasomes within the first 7 days of infection, leading to strongly altered proteasome activity (Khan et al., 2001). This suggests that CD8 T cell responses during the acute phase of viral and bacterial infection are primarily directed at immunoproteasome-dependent epitopes. Vaccines directed against epitopes that are poorly processed by the immunoproteasome would likely exhibit a less robust CD8 T cell response and not generate optimal protection against a particular pathogen.

Interestingly, immunoproteasomes assemble approximately four times faster than, and show greatly reduced stability relative to standard proteasomes (Heink et al., 2005). This suggests that immunoproteasome induction is a tightly regulated process, in which cytokines induced during the first few days of a viral infection signal a pressing need for immunoproteasome activity in the infected tissue. The relative instability of immunoproteasomes would provide a means for infected cells and tissues to quickly return to a normal state once immunoproteasomes are no longer needed, and it may suggest that ongoing or long-term immunoproteasome expression could actually be detrimental.

The role of the immunoproteasome during viral infection is still largely undefined, and there is evidence for organ-, virus-, and mouse strain-specific effects. Further studies are needed, especially with the newly generated triply deficient mice in which immunoproteasome activity is completely absent. Most studies examining immunoproteasome function during viral infection have focused almost exclusively on the effect of immunoproteasome subunits in shaping the repertoire of peptides available for MHC class I processing, and thus the hierarchy of CD8 T cell responses. However, the main function of the immunoproteasome during viral infection may actually be independent of the MHC class I antigen processing pathway. This is supported by the fact that B and T cells, which do not generally have a significant role as antigen-presenting cells (via MHC class I), express immunoproteasomes. A number of studies have suggested major roles for the immunoproteasome in T cell proliferation and survival, and there are hints from β1i^-/-^ mice that the immunoproteasome is also important for B cell development, as described above.

Accumulating evidence suggests that the immunoproteasome is critical for the removal of oxidized proteins and adaptation to oxidative stress (Ferrington et al., 2005, 2008; Kotamraju et al., 2006; Ethen et al., 2007; Hussong et al., 2010; Pickering et al., 2010). During coxsackievirus B3 (CVB3)-induced myocarditis, β5i^-/-^ mice developed more severe myocardial tissue damage compared to wild-type mice (Opitz et al., 2011). This was not due to a direct effect on viral replication. It is interesting to note that CD8 T cell responses in the heart, as measured by flow cytometry and immunohistochemistry, were equivalent or even slightly enhanced in β5i^-/-^ mice after CVB3 infection, suggesting that severe tissue damage in β5i^-/-^ mice was not due to an alteration in the CD8 T cell response. Rather, cardiomyocytes and inflammatory cells from β5i^-/-^ mice showed increased accumulation of polyubiquitinated protein conjugates and oxidant-damaged proteins following treatment with IFN-γ. Hearts from CVB3-infected β5i^-/-^ showed significant apoptotic cell death compared to infected wild-type mice. These findings suggest that the immunoproteasome protects cells from cytokine-induced proteotoxic stress by removing polyubiquitinated or oxidant-damaged proteins. Whether this role for the immunoproteasome is unique to CVB3-induced myocarditis or can be applied to other viral infections and disease states is unknown.

A recent study has suggested a new role for immunoproteasomes in maintaining cellular homeostasis (Raule et al., 2014b). Raule et al. (2014b) demonstrated that 26S immunoproteasomes degrade basic proteins at four- to sixfold higher rates compared to 26S standard proteasomes. This effect is observed specifically for proteins with a basic isoelectric point (high content in lysine and arginine residues), and not for neutral proteins. Histones, in particular, are extremely basic. Stimulation of cells with proinflammatory cytokines induces transcription of hundreds of genes through multiple regulatory pathways (Boehm et al., 1997; Schroder et al., 2004). Accumulation of free histones released from these sites of transcription could result in genomic instability and transcriptional inhibition (Singh et al., 2009). The ability of immunoproteasomes to remove excess free histones more efficiently than standard proteasomes could be an important mechanism by which immunoproteasomes maintain cellular homeostasis under conditions of stress and inflammation. This also suggests an additional reason for why CVB3-infected β5i^-/-^ mice have increased cellular damage and apoptotic cell death in heart tissue compared to wild-type mice. Perhaps β5i^-/-^ mice are unable to cope with the combined accumulation of oxidant-damage proteins and excess free histones in response to cytokine-induced stress and transcriptional activation.

Few studies have examined the effect of immunoproteasome deficiency on inflammation and protection of cells from virus- or cytokine-induced death during viral infection. It would be interesting to extend the studies described above with influenza, MCMV, LCMV, or other viruses in order to assess the role of the immunoproteasome in other aspects of the inflammatory response besides the generation of virus-specific epitopes for CD8 T cell responses.

## PATHOGEN INTERACTION WITH THE IMMUNOPROTEASOME

Components of many pathogens have been shown to interact with the immunoproteasome pathway. Perhaps not surprisingly, many of these pathogens establish chronic or persistent infections. Interference with the immunoproteasome pathway may be a common mechanism by which these pathogens inhibit CD8 T cell responses, either during acute infection (to facilitate the establishment of persistence) or during long-term persistence for ongoing evasion of the immune system.

HIV-1 inhibits immunoproteasome function, likely by a number of mechanisms (Haorah et al., 2004). Expression of viral p24 downregulates PA28β, β2i, and β5i in a DC line (JAWS II) and primary DCs. Exposure of those cell lines to HIV-1 p24 leads to a decrease in antigen presentation that can be overcome by pretreatment of cells with IFN-γ (such that the immunoproteasome is already upregulated by the time of p24 addition; Steers et al., 2009). HIV-1 Tat protein interacts with six β subunits of the standard 20S proteasome, as well as the immunosubunits β2i and β5i, to decrease catalytic activity (Apcher et al., 2003). Tat also binds to two α subunits, α4 and α7, preventing interaction of PA28 with the 20S core (Huang et al., 2002). The hepatitis C virus (HCV) non-structural protein NS3 directly binds to β5i and reduces immunoproteasome activity (Khu et al., 2004). Downregulation of immunoproteasome protease activity has been suggested as a mechanism by which HCV could interfere with processing of viral antigens for presentation on MHC class I and could avoid host immune surveillance during persistent infection.

Human adenovirus E1A interacts with the immunoproteasome subunit β2i, but not its constitutive counterpart β2. E1A (either in the context of adenovirus infection or via overexpression of E1A in the absence of other viral genes) also prevents IFN-γ-induced upregulation of immunoproteasome subunit expression by interfering with STAT1 phosphorylation (Berhane et al., 2011). Of note, adenoviruses have developed many other pre- and post-translational strategies to interfere with MHC class I processing and presentation that are independent of direct interactions between viral proteins and immunoproteasome subunits (reviewed in Blair and Blair-Zajdel, 2004).

Both human cytomegalovirus (HCMV) and MCMV inhibit IFN-γ-induced immunoproteasome formation in fibroblasts *in vitro* (Khan et al., 2004). Inhibition of immunoproteasome formation occurs at a pretranscriptional level, because transcriptional upregulation of PA28α/β, as well as all three immunosubunits, is impaired by infection. When cells are infected with an MCMV virus lacking M27, a gene that encodes a STAT2 inhibitor that interferes with IFN-γ receptor signaling, immunoproteasome expression is no longer inhibited.

## CONCLUSION

CD8 T cells often play significant roles during viral infection. In endogenous antigen presentation, the proteasome is crucial for the generation of antigenic peptides for binding to MHC class I and promoting CD8 T cell responses. The immunoproteasome is a specialized type of proteasome with altered peptide cleavage properties that is constitutively expressed in hematopoietic cells and induced in non-immune cells under conditions of inflammation. Evidence suggests that the immunoproteasome may play an important role during viral infection through regulation of CD8 T cell responses, activation of the NF-κB pathway, and management of oxidative stress. Many viruses have mechanisms of interfering with MHC class I processing, including direct interaction of viral proteins with immunoproteasome subunits. It is essential to better understand the role of the immunoproteasome in different cell types, tissues, and hosts in the context of diverse inflammatory states. An improved understanding of the mechanisms of immunoproteasome function could aid in the development of vaccines and treatment strategies for viral infections.

## Conflict of Interest Statement

The authors declare that the research was conducted in the absence of any commercial or financial relationships that could be construed as a potential conflict of interest.
